# A phase II RCT and economic analysis of three exercise delivery methods in men with prostate cancer on androgen deprivation therapy

**DOI:** 10.1186/s12885-015-1316-8

**Published:** 2015-04-25

**Authors:** Shabbir MH Alibhai, Daniel Santa Mina, Paul Ritvo, Catherine Sabiston, Murray Krahn, George Tomlinson, Andrew Matthew, Roanne Segal, Padraig Warde, Sara Durbano, Meagan O’Neill, Nicole Culos-Reed

**Affiliations:** 1 University Health Network, Toronto, ON M5G 2C4 Canada; 2 University of Toronto, Toronto, ON M5S 2J7 Canada; 3 University of Guelph Humber, Toronto, ON M9W 5L7 Canada; 4 York University, Toronto, ON M3J 1P3 Canada; 5 University of Ottawa, Ottawa, ON K1N 6N5 Canada; 6 University of Calgary, Calgary, AB T2N 1N4 Canada; 7 Tom Baker Cancer Centre, Calgary, AB T2N 4N2 Canada; 8 Toronto General Hospital, 200 Elizabeth St Room EN14-214, Toronto, ON M5G 2C4 Canada

**Keywords:** Prostate cancer, Androgen deprivation therapy, Exercise, Randomized controlled trial, Quality of life, Fatigue, Physical fitness, Cost-effectiveness, Patient adherence

## Abstract

**Background:**

Androgen deprivation therapy is commonly used to treat prostate cancer, the most common visceral cancer in men. However, various side effects often worsen physical functioning and reduce well-being among men on this treatment. Based on existing evidence, both resistance and aerobic training provide benefits for this population yet adherence rates are often low. The method of exercise delivery (supervised in-center or home-based) may be important, yet few studies have compared different models. Additionally, long-term exercise adherence is critical to achieve sustained benefits but long-term adherence data and predictors of adherence are lacking. The primary aim of this phase II, non-inferiority randomized controlled trial is to determine whether three exercise training delivery models are equivalent in terms of benefits in quality of life and physical fitness in this population. Secondary aims include examination of long-term adherence and cost-effectiveness.

**Design:**

Men diagnosed with prostate cancer, starting or continuing on androgen deprivation therapy for at least 6 months, fluent in English, and living close to one of two experienced Canadian study centers are eligible. Participants complete five assessments over one year, including a fitness assessment and self-report questionnaires. Socio-demographic and clinical data collection occur at baseline, bone mineral density testing at two time points, and blood work is performed at three time points. Participants are randomized in a 1:1:1 fashion to supervised personal training, supervised group training, or home-based smartphone- and health coach-supported training. Each participant receives a detailed exercise manual, including illustrations of exercises and safety precautions. Participants are asked to complete 4 to 5 exercise sessions per week, incorporating aerobic, resistance and flexibility training. Participant intensity levels will be monitored. The intervention duration is 6 months, with 6 months additional follow-up. Outcomes include: body composition, fitness testing, quality of life and fatigue, biological outcomes, and program adherence. Cost information will be obtained using patient diary-based self-report.

**Discussion:**

The goals of this study are to gain a better understanding of health benefits and costs associated with commonly used yet currently not compared exercise delivery models as well as an increased understanding of adherence to exercise.

**Trial registration:**

The trial has been registered at clinicaltrials.gov (Registration # NCT02046837), registered January 20^th^, 2014.

## Background

Prostate Cancer (PC) is the leading cancer diagnosed in North American men [1,2]. With improvements in early diagnosis and treatment, survival has been steadily increasing for this population [2]. One common treatment for men diagnosed with PC is androgen deprivation therapy (ADT), which is estimated to be used in 45% of men with PC [3,4]. Common side effects from ADT are fatigue, decreased muscle strength and cardiorespiratory function, loss of sexual functioning, mood changes, changes in body composition and negative effects on health-related quality of life (QOL) [5-8]. These changes often lead to a decrease in overall physical functioning and individual well-being [6,9]. Modifiable factors that can limit the burden of ADT need to be identified.

Exercise in the form of both resistance and aerobic training provide benefits for individuals with PC on ADT. In a randomized controlled trial (RCT), Segal et al. [10] found improvements in fatigue in the exercise group compared to a worsening of fatigue in the control group. Improvements in QOL were also noted following this in-center, structured exercise program [10]. Galvao et al. [11] found improvements in muscle strength following a 20-week resistance and endurance training program [11]. Improvements in 6-minute walk tests, stair climbing and chair rise fitness measures were also observed. In another study, Galvao et al. found significant improvements in the general health and physical health composite scores on the Medical Outcomes Study 36-item questionnaire (SF-36) for participants who completed a 12-week aerobic and resistance exercise program, compared to usual care [12].

These trials and others have been included in multiple systematic reviews of exercise interventions for individuals on ADT for PC. These reviews have found that, generally, exercise is safe and feasible, and exercise interventions are associated with multiple benefits for this population [9,13-16]. For example, Baumann et al. concluded that exercise interventions led to improvements in fatigue levels, muscular strength, flexibility, aerobic fitness, QOL, and well-being [9]. Santa Mina et al.’s review also found similar results regarding physical functioning and QOL outcomes with the additional finding that exercise program supervision by a certified professional was an important factor in achieving greater health benefits [14]. Altogether, findings from these reviews provide strong evidence of exercise benefits for patients on ADT.

Of interest, Thorsen et al. noted that in two of the studies reviewed, older age was associated with lower levels of participation intention [13]. This may be particularly important since PC is predominantly an older person’s disease, and aging itself is associated with multiple functional declines and sarcopenia [17].

While the most consistent benefits of exercise interventions in men on ADT have been seen with 1:1 supervised programs [15], few studies have directly compared exercise delivery methods. The systematic review by Keogh et al. noted significantly greater improvements in outcomes (physical functioning and QOL) for individuals in group-based supervised training vs. home-based training [15]. However, three important things should be noted. First, the noted improvement with group-based training compared to home-based training was based on indirect study comparisons. Second, home-based training provided significant pre-post increases in aerobic endurance and reduced fatigue. Third, only one RCT has directly compared multiple exercise delivery methods, comparing only 1:1 and group supervised training, but with a small sample size and without a home-based arm [18]. Because in-center, supervised, exercise programs are resource-intensive (and largely inaccessible), the demonstration of similar efficacy in home-based programs to these supervised programs would have important health service delivery implications and economic benefits and may help expand exercise delivery options for men on ADT.

It is also clear that the benefits obtained from an exercise program are maintained only as long as the exercise is maintained; therefore ensuring long term adherence to exercise programming has significant health implications. In RCTs of exercise in PC, adherence responses to exercise interventions are moderate to high but vary by protocol design. Supervised exercise intervention adherence ranged from 79% to 95% (as assessed by attendance at scheduled sessions) in three studies [10,19,20], whereas in a study of group-based training the attendance-based adherence was 94% [11,12]. In three home-based studies, the adherence ranged from 77% to 100% (assessed by way of patient reporting, booster session attendance or weekly group session attendance) [21-23]. In spite of a moderate to high level of adherence for exercise intervention across multiple delivery approaches, only Culos-Reed et al. [21] directly measured changes in activity levels (decreased in strenuous physical activity, however mild and moderate physical activity stayed relatively the same) from post-intervention to subsequent follow-up assessment. Additionally, follow-up assessments of fitness outcomes following the post intervention assessment [20-23] are rarely reported. However, when reported longer-term benefits vary, such that there is evidence of maintenance of intervention-based improvements at 6 months follow up in one study [20] whereas no differences in fatigue levels were found from baseline to the end of a 4-week intervention and again 4 weeks later, between the control and exercise groups [23].Follow-up data post-intervention were not obtained or reported in four of the above studies [10,12,19,22].

Even though multiple rigorous studies have demonstrated improvements in several health outcomes with an exercise intervention, exercise in the oncology setting remains unfunded in most countries by health insurance programs (public and private) and organizations. This contrasts sharply with cardiovascular rehabilitation, which is publicly funded in many jurisdictions, in part due to more advanced, robust evidence demonstrating cost-effectiveness. Indeed, in today’s fiscally strained funding environment, there is an increasing emphasis on demonstrating value (or return) on investment. Demonstration of the cost-effectiveness of exercise programs for men on ADT could lead to larger scale implementation of these programs.

Thus, the primary aims of this phase II non-inferiority RCT are:

- To determine the feasibility of conducting a large multi-center non-inferiority RCT of three exercise delivery models in men with PC on ADT. Feasibility outcomes include participant recruitment, retention, adherence, outcome capture, and satisfaction;
- To obtain preliminary efficacy estimates for (a) group-supervised in-centre, and (b) home-based (smartphone-assisted) supported exercise programs, compared to (c) a 1:1 supervised in-center exercise program study for the clinical outcomes of QOL and physical fitness, and to select a primary outcome for a subsequent phase III trial;
- To examine adherence to exercise and predictors of adherence in each exercise group during the 6-month intervention and for 6 months after program completion;
- To determine the feasibility of conducting an economic analysis comparing exercise interventions and usual care.

## Methods

This trial is taking place at two experienced academic tertiary care Canadian centers – the Princess Margaret Cancer Centre (Toronto, Ontario), and the Tom Baker Cancer Centre, (Calgary, Alberta). Ethics approval has been obtained at both institutions. All study participants provide written informed consent prior to study enrolment. The trial has been registered at clinicaltrials.gov (Registration # NCT02046837).

### Study population/participants

Men diagnosed with histologically confirmed PC of any stage, starting or continuing on ADT for at least 6 months (or who remain biochemically castrate after stopping ADT), able to speak, read and write in English, and living in close proximity to either study center are eligible for the study. Each potential participant is screened with the Physical Activity Readiness questionnaire (PAR-Q+ or PARmed-X) [24] and/or receives physician approval. If an individual already meets the American College of Sports Medicine (ACSM) guidelines for moderate to vigorous physical activity (MVPA) or has a condition that would interfere with his ability to participate (e.g. severe arthritis limiting ambulation, major neuropsychiatric abnormality, severe visual/hearing loss, poorly controlled pain), he is excluded from the study.

### Recruitment

The research coordinator at each study site will oversee recruitment. Estimated time for the recruitment phase will be 9 months, with the aim of recruiting 100 participants total. Potential participants are screened through PC clinics (primarily urology or radiation oncology) at each site and approached based on initial medical record review by the research coordinator. Potential participants are provided with a brief description of the study, and given an opportunity to ask any questions. If they are interested in study participation, written informed consent is obtained and a baseline assessment will be scheduled.

### Assessments

Participants complete five assessments in total: at baseline, three months, six months (end of intervention), nine months, and twelve months. The nine- and twelve-month assessments provide follow-up information after completion of the active intervention. Blinded outcome assessments are conducted by a Certified Exercise Physiologist (CEP; Canadian Society of Exercise Physiology, CSEP) or Registered Kinesiologist.

### Baseline assessment

The baseline assessment consists of obtaining socio-demographic and clinical data as well as assessing fitness using measures of resting heart rate (HR), blood pressure (BP), oxygen saturation, body composition (body mass index, waist circumference and body fat percentage), upper-lower body strength testing, and cardiovascular testing (peak VO2). Participants also complete questionnaires (see below) and are scheduled for a bone mineral density (BMD) test (if one has not been completed in the last year) and blood work (fasting glucose, prostate-specific antigen (PSA), testosterone, hemoglobin, and cholesterol profile). Serum is also banked for ancillary studies on participants at the Toronto site. All measures are detailed below and summarized in Table 1.

**Table 1:** **Summary of study measures at specified time points**

Domain/Measure	Time required	T_0_: (Baseline)	T_1_: (3 mo.)	T_2_:6 mo. (End Int.)	T_3_: 9 mo. (3 mo. f/u)	T_4_:12 mo. (6 mo. f/u)
**Quality of life**						
FACT-G (primary)	8-10 min	●	●	●	●	●
FACT-P	4-5 min	●	●	●	●	●
FACT-F	5 min	●	●	●	●	●
**Physical Fitness**						
VO_2_Peak	20 min	●	●	●	●	●
Sit-to-Stand test	1 min	●	●	●	●	●
Grip Strength	1 min	●	●	●	●	●
**Biological Outcomes**	<5 min	●		●		●
Blood glucose						
Cholesterol profile						
PSA (safety)						
Testosterone						
Hemoglobin (covariate)						
**Body Composition**						
Bone mineral density	30 min*	●				●
Body composition$	5 min	●		●		●
**Adherence**						
Accelerometer	**-**	●	●	●	●	●
GLTEQ	<5 min	●	●	●		
Sessional attendance#	-					
**Adherence Predictor Variables**						
NEWS-A	10 min	●	●	●	●	●
HCCQ	5 min	●	●	●	●	●
BREQ2	5 min	●	●	●	●	●
ROPAS	5 min	●	●	●	●	●
PAB questionnaire	5 min	●	●	●	●	●
Sedentary Behaviours	5 min	●	●	●	●	●
**Cost-Effectiveness**						
Health questionnaire	5 min	●	●	●	●	●
EQ-5D	5 min	●	●	●	●	●
**Study Completion**	5 min					●

*Can be done on separate day to reduce participant burden.

$Includes waist circumference, waist circumference:height ratio, and % body fat using bioelectrical impedance analysis.

#Only for those in supervised groups (done weekly).

***Abbreviations****:BREQ2* Behavioral Regulations in Exercise Questionnaire – 2, *EQ-5D* EuroQol 5 dimensions of health scale, *FACT-G* Functional Assessment of Cancer Therapy General, *FACT-F* Fatigue subscale, *FACT-P* Prostate subscale, *GLTEQ* Godin Leisure Time Exercise Questionnaire, *HCCQ* Health Care Climate Questionnaire, *Int* Intervention, *NEWS-A* Neighborhood Environment Walkability Scale short form, *PAB* Planning, Attitudes, & Behavior questionnaire, *PSA* prostate-specific antigen, *ROPAS* Relatedness to Others in Physical Activity scale, *VO2 Peak* Peak Volume of Oxygen Consumption.

### Assessments (three, six, nine and twelve months)

All follow-up assessments include updates on clinical data, fitness measurements, and self-report questionnaires. BMD testing occurs only at baseline and twelve months, blood work and body composition testing is done only at baseline, six months and twelve-months (Table 1).

### Randomization

Participants are allocated to treatment groups following the baseline assessment through a computer-generated stratified randomization scheme developed by the study biostatistician (GT) and administered through an independent website. Although the CEP outcome assessors are blinded to treatment assignment, for practical reasons participants and the intervention CEPs cannot be blinded. Participants are randomized equally to one of three groups: 1:1 supervised training, supervised group training, and home-based smartphone-assisted training. Randomization is stratified by duration of prior ADT use (<3 months versus 3 months or more), as evidence suggests that muscle changes and impact on QOL is most appreciated within the first 3–6 months of ADT use [6] and exercise response may be partially modified by duration of ADT [25].

Following randomization, participants are scheduled to attend their first session (1:1 or group-based training) or an orientation session (delivered by the study coordinator) for home-based training. All participants will receive a study manual at this time point.

### Objective measures/ Primary and secondary outcomes

#### Body composition

Body composition is measured following the CSEP’s [26] Canadian Physical Activity Training for Health [27] assessing waist circumference (WC), weight, waist to hip ratio (WHR), body mass index (BMI). Body fat percentage (BF%) is measured via bioelectrical impedance analysis (BIA) using the Tanita TBF-300A device (Illinois, USA). Additionally, participants receive a BMD scan of the lumbar spine, total hip, and femoral neck using dual x-ray absorptiometry.

### Fitness testing

Prior to the start of fitness testing, resting HR, BP, and oxygen saturation are measured. Aerobic fitness is directly assessed through the modified Bruce treadmill protocol (treadmill-based graded exercise test) [28], which assesses volitional peak oxygen consumption (VO_2_ peak) using a metabolic cart (Parvo Medics TrueOne 2400 Metabolic Measurement System). Lower body strength is measured with a one minute sit-to-stand test [29,30] and grip strength is used to assess upper body strength. Grip strength is measured with a Jamar dynamometer averaging three readings obtained in each hand [31].

### QOL and fatigue

Functional Assessment of Cancer Therapy – General (FACT-G) is used to assess general health-related QOL [32], whereas the FACT-P (Functional Assessment of Cancer Therapy - Prostate) evaluates prostate-specific QOL [33] and the FACT–F (Functional Assessment of Cancer Therapy – Fatigue) is used to evaluate cancer-related fatigue [34]. All three questionnaires are validated and have been widely used in cancer research measures, and in particular, in RCTs of exercise in men on ADT [14].

### Biological outcomes (blood data collection)

At three assessment time points (baseline, 6 months and 12 months), participants’ blood is drawn to analyze fasting blood glucose, lipid profile (total cholesterol, low density lipoprotein, high density lipoprotein, and triglycerides), testosterone, as well as PSA levels. Blood glucose and lipids are being monitored as ADT is associated with an increased risk of both diabetes and dyslipidemia [35,36]; conversely, exercise is associated with improvements in both metabolic parameters [37]. PSA levels are being monitored as a safety measure [38], as in other exercise trials [10,12,39], and testosterone is measured to assess adequacy of castration.

### Adherence

Program adherence and predictors of adherence are assessed. Adherence measures include physical fitness outcomes (as the most proximal, objective outcome of exercise adherence), accelerometry, as well as attendance at exercise sessions (for those in supervised programs). For those in the home-based, smartphone-assisted arms, use of the self-report software (Connected Wellness Platform, NexJ Systems, Toronto, Canada) and interview responses (obtained by health coaches) will provide proxies for attendance and exercise program engagement. Accelerometers (Actigraph GT3X, Pensacola, FL) will be worn for seven days at each assessment time point. Accelerometers will be worn during waking hours, allowing for capture of total physical activity during the observation period [40]. Data will be extracted from the accelerometer in 60-second epochs and will be screened through; (i) at least 4 days of valid data, including (ii) at least 10 hours of wear time per day; (iii) non-wear time will be assessed as periods of time with no movement (0 counts per minute) for more than one hour at a time.

Two common definitions of adherence are adopted: 1) at least 150 minutes of MVPA per week, based on the ACSM guideline for cancer survivors [28] and the revised CSEP guideline for adults and older adults [41]; and 2) at least 10,000 steps per day [42] for 6+ days per week. In a sensitivity analysis, a recently described age-specific cut-off of 7,500 steps per day for older adults [43] is also tested. The accelerometer data will be analyzed by examining the total amount of MVPA and sedentary behavior during the 7 days. MVPA is defined as activity >1952 counts per minute [42] and sedentary behaviour as <100 counts per minute and time spent inclined.

**Figure 1:**
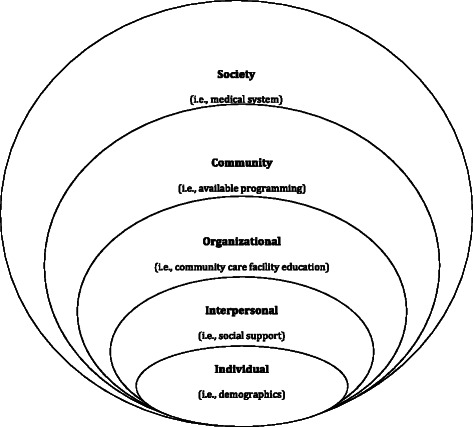
Social ecological framework for understanding exercise determinants.

Predictors of adherence are targeted based on a social ecological framework (Figure 1) [44]. Multiple potential determinants of exercise adherence and the inter-relatedness of these determinants are examined using brief validated measures at each level: exosystem (Neighborhood Environment Walkability Scale: Short Form (NEWS) [45]); mesosystem (Health Care Climate Questionnaire [46] and Related to Others in Physical Activity Scale [47]); and microsystem (Behavioral Regulations in Exercise Questionnaire-2 [48,49] and a Planning, Attitudes, & Barriers scale [50,51]).

### Intervention

The six-month exercise intervention consists of one of three exercise delivery arms; supervised 1:1 personal training, supervised group training and unsupervised home based (smartphone-assisted) training. In all three intervention arms, each participant meets with a CEP to receive instructions regarding the exercise program and orientation to a detailed exercise manual that includes illustrations of exercises and safety precautions for exercising. All participants are asked to complete 4–5 days per week of mixed modality exercise incorporating aerobic, resistance and flexibility training, all training programs are prescribed based on the FITT principle: Frequency, Intensity, Time and Type. Each program includes an education component of 12 topics that will focus on common concerns facing new exercisers (see Table 2). This occurs during their sessions or weekly phone calls throughout the intervention period, administered by the CEP for the 1:1 supervised and group-based training, and by health coaches for home-based participants. Both group and 1:1 supervised training occur under the supervision of a CEP. Participants’ programs are individualized based on their baseline fitness assessment, with the target time and absolute workload (target heart rate: 60-70% of heart rate reserve) kept the same across all interventions. Relative intensity is maintained throughout the program; therefore, participants are progressed similarly to ensure this occurs (see Progression section below).

**Table 2:** **Education topics**

Education topics	Key points
1) Introduction to Exercise	• Benefits of Physical Activity
	• Program targets 3 areas of PA (aerobic, resistance, and flexibility)
	• PA is safe, feasible and has shown to provide benefits
2) Goal Setting	• Goal setting will assist with your dedication and motivation to complete the exercises
	• SMART Goals - Specific, Measurable, Attainable, Realistic, Timely
	• Use the goal worksheet in the manual
	• Make long term and short term goals
3) Behavior Change	• The plan you set out may not be followed 100%
	• Anticipate obstacles that may come as you are changing a behaviour and develop strategies for dealing with it before it arises
	• Monitor your progress, Reward yourself, Visualize your success
4) Planning for Barriers	• Biggest perceived obstacles
	○ Lack of time, self-discipline, partner and ability
	• Plan ahead for periods of inactivity
5) Social Support	• You are more likely to be successful if your family, friends and even co-workers are supportive of you
	• Social support can occur in many forms – encouragement, completing activities with you, etc.
6) Monitoring Behavior	• Mix up your activities to stay motivated
	• Try something new, or something you have done previously
	• It is very easy to enter an exercise rut
7) Maintaining Motivation	• Greatest source of motivation: Fun/enjoyment/stimulation, feeling of accomplishment, pleasure of learning and benefits (i.e. improved sleeping)
	• Pursue something that you enjoy, that is convenient to your schedule
	• Take opportunities to be active
8) Personal Control	• Believing that you are in control of your own life give you reinforced motivation and further commitment to make changes
9) Self- Discipline, Reward & Attitude	• Self-discipline can result in increased productivity, improved self-esteem and confidence
	• Rewards – use workbook in manual
	• Attitudes toward change can determine whether you will be successful
10) Adapting your Program	• Adapting your program – FITT principle
11) Health and the Media	• Be mindful of the ‘Get fit quick’ media marketing – Health eating and regular PA will help maintain a long-term health lifestyle
12) Lifelong Active Living	• Use some of the tips and tricks in the manual to assist with continuing your active life
	• Change things up, work towards small goals, work with a friend, etc.

The 10-point Rating of Perceived Exertion (RPE) scale is used to monitor exercise intensity levels [52], with participants instructed to maintain their intensity level at 3–6 during exercise sessions. RPE levels are related to target HR, as it corresponds to the linear change in HR [53]. HR monitors (Polar, NY, USA) are used at 3-week intervals during the exercise sessions across all intervention arms. This is to ensure participants maintain appropriate heart rate levels during the session, ensuring calibration with the 3–6 rating on the RPE scale throughout the intervention period. Home-based participants are trained to use HR monitors which are provided to them. Participants in the supervised 1:1 and group programs will have staff present to assist in HR measurement.

Study staff will hold monthly conference calls and staff will be trained on testing and training procedures, to ensure standardization across both sites. Supervised exercise sessions are documented with standard forms at all sites.

### Progression

Participant progression is individualized, and monitored by a CEP or health coach every three weeks to ensure that progression is occurring. The participant’s intensity level during the exercises sessions (both aerobic and resistance) will be used as an indicator of whether the participant is ready to progress. CEPs or health coaches adjust intensity levels to ensure participants maintain the desired intensity range throughout each exercise session. Documentation of this progression is completed using a standard form across all arms of the intervention and both study sites. Participants who are working below their target HR when performing aerobic exercise will first increase their exercise duration (i.e., from 15 to 20 minutes), followed by an increase in exercise intensity (i.e., increase walking speed or grade). Similarly, participants who are able to complete ≥12 repetitions and 3 sets of a specific resistance exercise will increase the resistance (i.e., blue band to purple band; next resistance level) they are performing the exercise at to ensure that they are continuing to progress effectively throughout the program.

### 1:1 supervised training

Participants in the 1:1 supervised training complete three sessions per week with a CEP for a period of six months, and are encouraged to perform one to two additional weekly independent (home-based) sessions. Each exercise session consists of cardiovascular training for 15 to 30 minutes, resistance training exercises (working major muscle groups), and flexibility training (including 5–10 minutes of stretching at the end of each session). All participants are provided with resistance bands for home use.

### Group supervised training

This protocol differs from the 1:1 training protocol in only one way: a group of 4–6 individuals are supervised by a CEP at each session.

### Home-based training

The same protocol and training frequency as the 1:1 and group supervised training program is explained and recommended to be followed by the home-based smartphone-assisted participants. Along with the resistance bands provided, participants in the home-based training group also receive a stability ball, an exercise mat, a HR monitor (with instructions), and a smartphone with a 6-month paid phone and data plan. Participants in the home-based arm complete an orientation session with a CEP prior to the commencement of the training. Participants are oriented to the same exercise manual that is provided to the supervised group participants, and review the resistance and flexibility exercises with a CEP to ensure proper technique. During the orientation participants also meet with the health coach to review the smartphone application and the role of the health coach. Remote health coaches and smartphone technology are intended to provide support to participants in the home-based training program during the intervention phase, as there is no direct (i.e. face-to-face) supervision with this group. The health coach connects with participants on a regular basis (ideally once each week) to follow up on the week’s exercise sessions and to provide guidance for the participants and assist with any smartphone application issues. Health coaches instruct the participant when to use the HR monitors to evaluate intensity every three weeks. A customized smartphone application is used (Connected Wellness Platform) that allows users to input health information, levels of symptoms, and exercise routines, as well as tracking progress over time.

### Tapering

#### Supervised sessions

Tapering allows participants within the supervised training arms to transition from predominately supervised training to independent exercise training. This is in place to encourage participants to continue to exercise independently at the same intensity and frequency as achieved during their supervised exercise programs. Tapering occurs for participants in the 1:1 supervised and group-based training arms. Starting in month five, supervised sessions are reduced to two sessions per week. In the final month (month six), supervised sessions are tapered further to one session per week. Participants are encouraged to replace the supervised sessions with equivalent independent exercise sessions.

### Health coach support

For the home-based group, a similar concept of tapering will be introduced to reduce the level of support provided by health coaches. In the first four months the health coaches will contact each participant once every week and will be available seven days per week, with turnaround time within an hour. In the final two months of the intervention, the health coaches will contact each participant once every two weeks and will be available five days a week. Text message turnaround time will be reduced from within the hour to next day.

### Safety

The CEP reviews with each participant precautions and safety while exercising during the participant manual orientation and participants are reminded to review the manual prior to commencing independent exercise sessions. All exercise trainers have cardiopulmonary resuscitation (CPR) and automated external defibrillator (AED) training and have received safety training at each site. Any adverse events are document using the National Cancer Institute common terminology criteria for adverse events v4.0 [54].

### Cost-effectiveness analysis

This phase II study investigates the feasibility of completing a phase III trial and a full companion economic evaluation. In the phase II design there will be no formal cost-utility analysis, however outcomes relevant to economic analyses regarding health status will be collected at each assessment time point using a generic utility instrument (the European QOL 5-dimension measure (EQ-5D)) [55]. Costs are captured with a patient-reported diary that has been used in prior studies of men with PC [56,57]. Costs related to health care utilization (e.g. visits to a physician or physiotherapist), transportation, purchase of exercise equipment (including gym membership fees), and related costs are captured at each of the five time points where QOL and fitness data are captured.

### Sample size calculation/power

Following standard guidelines for a phase II RCT [58-60], we have targeted 30 patients per arm (90 patients in total) to provide precise estimates of parameters related to the primary outcomes as well as important feasibility information that will be crucial to inform the phase III study. Assuming 10% drop-out, we will recruit 100 patients across both sites.

### Statistical analysis

Statistical analysis focuses on feasibility outcomes and those which will inform decisions about moving forward to and planning a phase III clinical trial. Feasibility outcomes include recruitment rates, and proportions of participants retained and adherent at landmark times throughout the study, all of which will be estimated with 95% confidence intervals. Additionally tallies are kept on reasons for not participating. Each participant also completes an exit interview and satisfaction survey which will be analyzed for participant satisfaction within the study and cost effectiveness analysis.

Adherence is assessed through the two different binary definitions given previously, based on steps or hours of exercise. We estimate proportions adhering to exercise in each three-month period and use generalized estimating equation logistic regression models to compare adherence between groups and assess predictors of adherence over the course of the study. Predictors include sociodemographic (e.g. age, education), clinical (disease stage), baseline fitness, and ecological variables (NEWS, etc.).

Quality of Life (FACT-G and FACT-P), musculoskeletal fitness, and aerobic fitness are efficacy outcomes that are analyzed using linear mixed effects model with subject-specific random effects and group-by-time interactions. The analysis will not focus on differences between groups, but, to assist with phase III trial design, estimation of measures of within and between-person variance.

As previously mentioned, there is no formal cost-effectiveness analysis, however data obtained (diary and EQ-5D completion and participant acceptance) is used to help develop this outcome for the phase III study. Descriptive estimates of costs by category and per intervention arm as well as per site (Toronto, Calgary) will be calculated.

### Criteria to move forward to phase III RCT

To move forward to the phase III intervention, we have identified pre-specified criteria which must be met during the phase II study. Reasonable recruitment (at least 25% of potentially eligible approached participants), adherence (70%) and retention rates (70%), at least moderate satisfaction of participants, and capture of at least 80% of clinical outcomes collected are the minimum criteria. The intention is that all three of the interventions will move forward to the phase III study; however, if one arm proves to be inferior to the other two, based on adherence, retention, satisfaction or QOL and physical fitness results, then only two will move to phase III.

## Discussion

Increasing amounts of data from well-designed intervention studies have demonstrated statistically and clinically significant gains in fitness and QOL outcomes with exercise-based interventions [16]. The most robust data support 1:1 supervised programs, but these are resource-intensive and largely inaccessible to most patients. Whether group-supervised or home-based programs, both of which are likely significantly less resource-intensive and more feasible to implement, are equally effective as 1:1 supervised programs remains unclear. Altogether, wider adoption of exercise programs is important as the status quo of the majority of men treated with ADT is that they remain physically inactive and at risk of multiple potentially avoidable side effects [13,61,62]. In addition, longer-term adherence, after completion of acute training programs, is key to ensuring ongoing health benefits, yet few data have been published examining longer-term follow-up adherence with any exercise delivery model. Moreover, little is known about factors predicting long term adherence; such information can be used in enhancing longer-term exercise programming effects. Hence, the goals of this study are a better understanding of health benefits (QOL, physical fitness, and other clinically relevant endpoints) provided through three varying exercise delivery models that include evaluation of the cost-effectiveness of program provision (for men on ADT for PC), and adherence associated with each approach (during and after the formal six-month intervention period).

The current study is designed to address important limitations in prior studies and advance the science of exercise delivery for men on ADT. First, this study is directly comparing the three main exercise delivery models to identify whether less resource-intense approaches are as efficacious as the reference standard of supervised personal training. Second, adherence will be carefully studied during and, more importantly, after the formal six-month intervention period. Moreover, insights will be gained into enabling factors and barriers to exercise adherence. Third, we will formally begin to evaluate the cost effectiveness of each exercise delivery model; such information should ultimately inform health policy. Additional innovations include structured exercise tapering (to facilitate independent exercise), introduction of a smartphone app and health coach support to enhance home-based exercising, and other features.

The evidence-based knowledge gained with this research will provide an understanding of the best (clinically effective, high adherence, cost effective) exercise program for this population and will help strengthen research in this field. Although the results from the phase II trial will not be definitive, this step is foundational and the knowledge that will be generated will be incorporated into the design and execution of a multi-center phase III RCT.

## Abbreviations

ACSM: American College of Sport Medicine
ADT: Androgen Deprivation Therapy
AED: Automated external defibrillator
BIA: Bioelectrical impedance analysis
BMD: Bone mineral density
BMI: Body mass index
BP: Blood Pressure
CEP: Certified Exercise Physiologist
CPAFLA: The Canadian Physical Activity, Fitness and Lifestyle Approach
CPATH: Canadian Physical Activity Training for Health
CPR: Cardiopulmonary resuscitation
CSEP: Canadian Society of Exercise Physiology
FACT-F: Functional Assessment of Cancer Therapy - Fatigue
FACT-G: Functional Assessment of Cancer Therapy - General
FACT-P: Functional Assessment of Cancer Therapy - Prostate
FITT: Frequency, Intensity, Time, Type
HR: Heart Rate
MVPA: Moderate to Vigorous Physical Activity
PARmed-X: Physical Activity Readiness Medical Examination
PAR-Q: Physical Activity Readiness Questionnaire
PC: Prostate Cancer
PSA: Prostate-specific antigen
QOL: Quality of Life
RCT: Randomized Controlled Trial
RPE: Rated perceived exertion
SF-36: Short Form 36, Medical Outcomes Study Quality of Life questionnaire
WC: Waist circumference
